# The improved anticancer effects of Bortezomib-loaded hollow mesoporous silica nanospheres on lymphoma development

**DOI:** 10.18632/aging.202146

**Published:** 2020-12-03

**Authors:** Jie Shen, Ruihuan Wang, Qing Wang, Minjuan Zhang, Chunyan Liu, Zhenxia Tao, Guohong Su

**Affiliations:** 1 Department of Hematology, Centre Hospital of Cangzhou, Cangzhou, Hebei Province, China; 2 Department of Central Laboratory, Centre Hospital of Cangzhou, Cangzhou, Hebei Province, China

**Keywords:** hollow mesoporous silica nanospheres, bortezomib, drug delivery, tumor-suppressing effect, lymphoma

## Abstract

As the first clinical proteasome inhibitor, Bortezomib (BTZ) has been reported to improve the outcome of lymphoma. However, due to the unstable property, low bioavailability, and hydrophobic properties of BTZ, it is needed to develop effective drug delivery systems to deliver BTZ into targeted cells or organs. Here we developed a bortezomib (BTZ)-loaded HMSNs (BTZ@HMSNs) system, which can sustain the release of BTZ in targeted tissues. *In vitro* assays showed that BTZ@HMSNs limited cell proliferation and augmented apoptosis of lymphoma SNK-1 cells. Moreover, BTZ@HMSNs significantly diminished migration and invasion of SNK-1 cells as compared with BTZ. In contrast to the upregulation of SHP-1, BTZ@HMSNs decreased the mRNA levels of *c-Kit*, *NF-κB*, and *JAK1*, which elicit oncogenic role in lymphoma development. Importantly, lymphoma mice model showed that BTZ@HMSNs significantly activated p53 signaling and reduced tumor volume and weight compared with free BTZ. Our data thus demonstrate that BTZ@HMSNs manifests improved tumor-suppressing effect *in vitro* and *in vivo* compared to free BTZ. We believe that HMSNs is a promising strategy for delivering therapeutic agents for cancer treatment.

## INTRODUCTION

As a common malignant tumor of the immune system, malignant lymphoma originates from the immunocyte-rich lymphoid tissues with high mortality, and is caused by the clonal expansions and differentiation of B, T, or NK cells during the immune response [1]. To date, the effective therapeutic methods for lymphoma are immunotherapy, sophisticated surgical resection and advanced chemoradiation [2, 3]. Despite the substantial progress in diagnosis and treatments of lymphoma, unsatisfactory prognosis and poor survival remain an issue due to high rates of invasiveness and recurrence [4]. Therefore, it is essential to search for new drugs and novel treatment methods for lymphoma.

It is well known that ubiquitin-proteasome system (UPS) exerts crucial roles in various cell biological process such as intracellular signal transduction, cell cycle and apoptosis by regulating cellular proteins degradation [5]. Inhibition of the proteasome has been widely reported to be a therapeutic target for various cancers [5]. Bortezomib (BTZ) is the first clinical proteasome inhibitor applied for the treatments of mantle cell lymphoma and multiple myeloma [6]. Previous studies have demonstrated that BTZ shows favorable overall survival and progression-free survival as well as durable responses in lymphoma [7–9]. However, the clinical use of BTZ is compromised by inefficient cancer treatment due to its unstable property, low bioavailability, and hydrophobic properties [6, 10]. Thus, it is required to develop effective drug delivery systems that are able to deliver BTZ into targeted cells or organs.

Currently, nanoparticles have been reported to be promising drug delivery systems [11, 12], and are widely applied into cancer therapy [13]. The hollow mesoporous silica nanospheres (HMSNs) as novel nanoparticles possess unique properties such as controllable surface and particle size, good stability, highly-hydrophilic nature, high mechanical strength, and favorable biocompatibility, compared with other nanomaterials, including liposomes, polymers and polymeric micelles [14–16]. In particular, HMSNs are able to load hydrophobic drugs by electrostatic adsorption, and higher drug loading capacity of HMSNs has been confirmed due to the suitable pore volume and the interstitial hollow space [17]. Previous study has shown that BTZ loaded nanoparticles can encapsulate free BTZ, and exhibit enhanced anti-cancer effect than free BTZ *in vitro* [18]. Notably, recent study has developed BTZ-loaded HMSNs (BTZ@HMSNs) with a high biocompatibility, and BTZ@HMSNs exhibit improved tumor-suppressing effects compared with free BTZ for non-small cell lung cancer therapy both *in vitro* and *in vivo* [19]. However, the effect and mechanism of the BTZ@HMSNs in lymphoma has not been investigated.

In the current research, the BTZ@HMSNs were successfully prepared, and then characterization and *in vitro* drug release of the BTZ@HMSNs were detected. In addition, the effects of the BTZ@HMSNs on cell proliferation, apoptosis, migration and invasion in lymphoma SNK-1 cells, as well as the potential mechanism were explored.

## RESULTS

### Preparation and characterization of the BTZ@HMSNs

**Figure 1:**
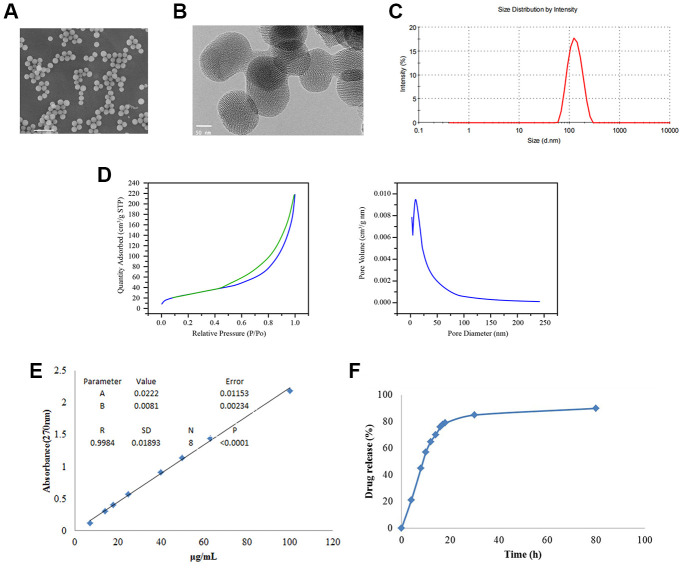
**Preparation and characterization of BTZ@HMSNs.** (**A**) The morphologies of BTZ@HMSNs by scanning electron microscope. Bar = 500 nm. (**B**) The morphologies of BTZ@HMSNs by transmission electron microscope. Bar = 50 nm. (**C**) Size distributions of BTZ@HMSNs determined by dynamic light scattering. (**D**) N2 adsorption–desorption curve (left) and pore distribution (right) of BTZ@HMSNs. (**E**) The standard curve of BTZ by plotting the absorption at 270 nm against BTZ concentrations. (**F**) Cumulative profiling of drug release of BTZ from the BTZ@HMSNs in phosphate-buffer saline (PBS, pH 7.4) at room temperature. BTZ, bortezomib; HMSNs, hollow mesoporous silica nanospheres

To improve the antitumor efficiency of Bortezomib (BTZ), we generated the Bortezomib-loaded hollow mesoporous silica nanospheres (BTZ@HMSNs). We then used scanning electron microscope (SEM) and transmission electron microscope (TEM) assays. As shown in Figure 1A, 1B, the average sizes of the BTZ@HMSNs were about 150 nm. Accordingly, DLS revealed that the hydrodynamic diameters of the BTZ@HMSNs were around 150 nm (Figure 1C). In addition, as shown in Figure 1D, the N2 adsorption–desorption isotherms and pore size distributions were 2-50 nm of pore size distributions with an average diameter of 12 nm, which suggested the typical hollow structure of BTZ@HMSNs. Besides, we also found that the LC of BTZ@HMSNs was 9.8%. We next examined the BTZ release from the BTZ@HMSNs. The absorbance at 270 nm of BTZ (ranged from 7-100 μg/mL) were measured, and a standard curve was created (Figure 1E), which showed a good linear relationship (r^2^ = 0.998). Cumulative drug release profile of BTZ@HMSNs revealed that almost 60% of BTZ was released from HMSNs within 10 hrs, about 30% of BTZ was released within 80 h with a sustained and slow release (Figure 1F).

### BTZ@HMSNs treatment inhibits the proliferation of lymphoma cells

**Figure 2:**
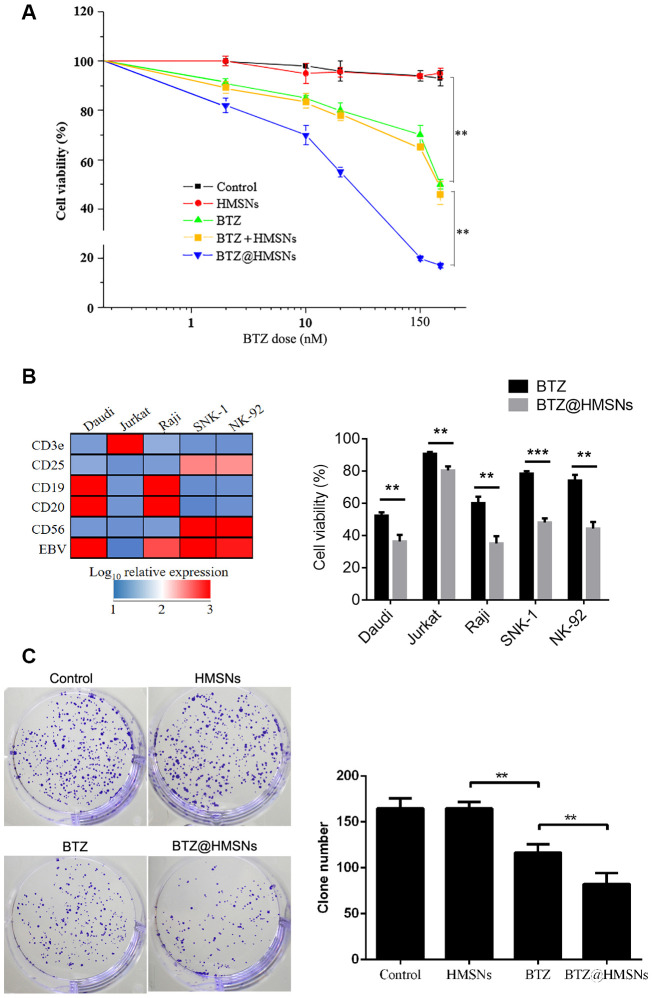
**BTZ@HMSNs restricts the proliferation of lymphoma.** (**A**) Cell viability of SNK-1 cells treated with PBS (control), HMSNs, BTZ (set from 1 to 150 nM), HMSNs+BTZ (containing 1-150 nM of BTZ) and BTZ@HMSNs (containing 1-150 nM of BTZ) by MTT assay. (**B**) The gene expression profile of Daudi, Jurkat, Raji, SNK-1 and NK92 cells by qRT-PCR (Left). Cell viability of indicated cells treated with BTZ or BTZ@HMSNs by MTT assay (Right). (**C**) Colony number of SNK-1 cells treated with PBS (control), HMSNs, BTZ, BTZ@HMSNs by colony formation assay. BTZ, bortezomib; HMSNs, hollow mesoporous silica nanospheres. The differences between groups were analyzed by one-way ANOVA followed by multiple comparison with Tukey test. **P < 0.01 and ***P < 0.001. Values are means ± SD.

To evaluate the cytotoxicity of BTZ@HMSNs, we assessed the proliferation of SNK-1 cells treated with PBS, HMSNs, BTZ, HMSNs+BTZ or BTZ@HMSNs. As shown in Figure 2A, in contrast to the little effects on cell viability by HMSNs or PBS, BTZ treatment exhibited suppressive effects on SNK-1 cell proliferation, which indicated that HMSNs was non-toxic to cells. Notably, compared with cells with BTZ, BTZ@HMSNs treatment significantly decreased cell viability in dose-dependent manner in SNK-1 cells (Figure 2A). Moreover, directly adding both HMSNs and BTZ in the medium (HMSNs+BTZ) had similar inhibition effect as BTZ alone, confirming that the increased suppressive effects of BTZ@HMSNs on SNK-1 cell proliferation was not due to the pre-released BTZ during incubation. To determine whether BTZ@HMSNs treatment also affects the activity of other lymphoma cells, we employed a series of lymphoma cells, including B cell lymphoma cell lines (Daudi and Raji), T cell lymphoma cell line (Jurkat) and NK cell lymphoma cell lines (SNK-1 and NK-92). In contrast to the EBV-negative Jurakt cells, Daudi, Raji, SNK-1 and NK-92 cells are EBV-positive cells (Figure 2B). We then used 20 nM BTZ or BTZ@HMSNs to treat lymphoma cells. As shown in Figure 2B, BTZ@HMSNs treatment elicited more suppressive effects on lymphoma cells, which supported the notion that the anticancer effects of BTZ was in an EBV-independent manner. In addition, colony formation assay further confirmed the significance of BTZ@HMSNs on inhibition of lymphoma cell proliferation (Figure 2C). Our data thus demonstrate that BTZ@HMSNs elicit improved suppressive effects on lymphoma cell proliferation.

### Effect of BTZ@HMSNs on cell apoptosis in SNK-1 cells

**Figure 3:**
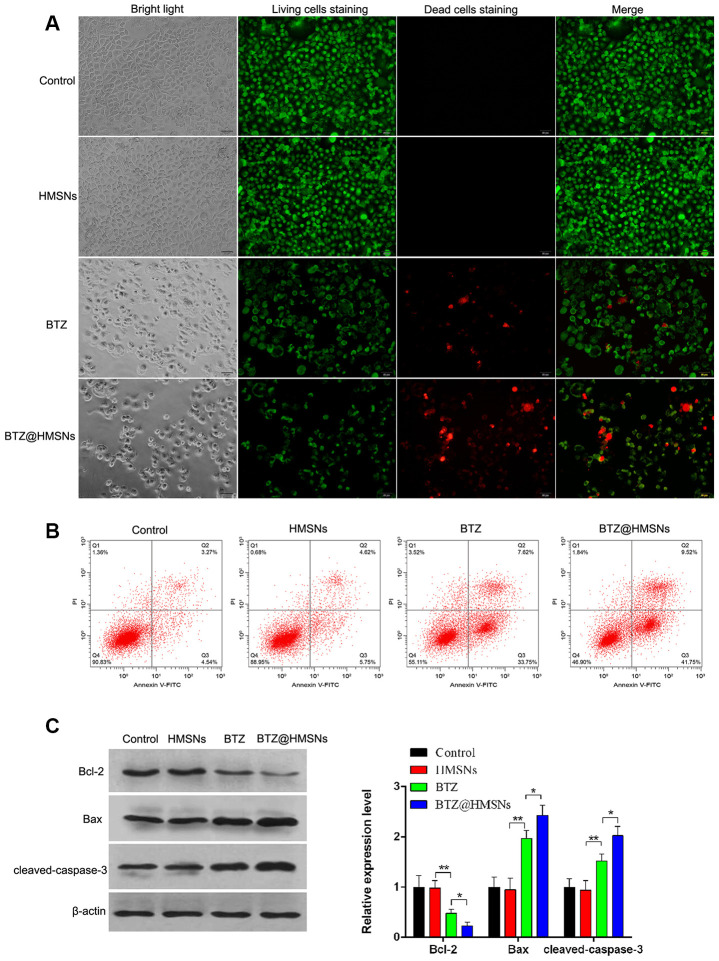
**BTZ@HMSNs augments cell death of lymphoma.** (**A**) Live/dead staining detection for SNK-1 cells treated with PBS (control), HMSNs, BTZ (75 nM), BTZ@HMSNs (containing 75 nM of BTZ). Scale bar equals 5 nm. (**B**) Cell apoptosis rate of SNK-1 cells treated with PBS (control), HMSNs, BTZ, BTZ@HMSNs by flow cytometry analysis. (**C**) The expression of apoptosis-related proteins (Bcl-2, Bax, and cleaved-caspase-3) in SNK-1 cells treated with PBS (control), HMSNs, BTZ, BTZ@HMSNs by western blotting. BTZ, bortezomib; HMSNs, hollow mesoporous silica nanospheres. The differences between groups were analyzed by one-way ANOVA followed by multiple comparison with Tukey test. *P < 0.05 and **P < 0.01. Values are means ± SD.

To assess the effectiveness of BTZ@HMSNs on apoptosis, we employed PI staining to assess the cell death induced by PBS, HMSNs, BTZ or BTZ@HMSNs. As shown in Figure 3A, cells with HMSNs showed few fluorescence intensities, which was similar to control cells. Meanwhile, compared with cells with HMSNs, increased fluorescence intensities were observed in cells with BTZ, and cells with BTZ@HMSNs had higher fluorescence intensities than that in cells with BTZ (Figure 3A). In addition, flow cytometry analysis showed few apoptotic cells were detected in control cells and cells with HMSNs, while the number of apoptotic cells was increased in cells with BTZ compared with cells with HMSNs. Notably, BTZ@HMSNs treatment increased the number of apoptotic cells as relative to cells treated with BTZ (Figure 3B). Consistently, western blotting showed that compared with cells with HMSNs, BTZ treatment distinctly inhibited Bcl-2 level as well as promoted the expression of Bax and cleaved-caspase-3 (*p* < 0.01), and BTZ@HMSNs further inhibited Bcl-2 level as well as promoted the expression of Bax and cleaved-caspase-3 (Figure 3C, *p* < 0.05). Our data thus demonstrate that the anticancer effects of BTZ@HMSNs are improved.

### The suppressive effect of BTZ@HMSNs on migration and invasion of SNK-1 cells

**Figure 4:**
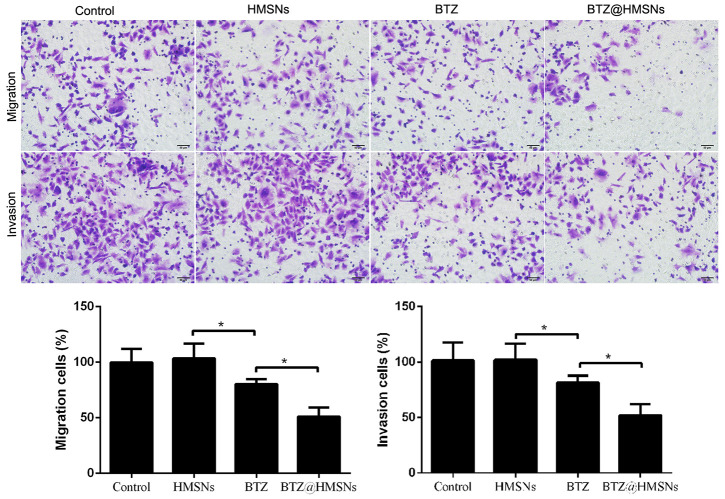
**Cell migration and invasion of SNK-1 cells treated with PBS (control), HMSNs, BTZ (75 nM), BTZ@HMSNs (containing 75 nM of BTZ) by Transwell assay.** Scale bar equals 5 nm. BTZ, bortezomib; HMSNs, hollow mesoporous silica nanospheres. The differences between groups were analyzed by one-way ANOVA followed by multiple comparison with Tukey test. *P < 0.05. Values are means ± SD.

We next employed Transwell assay to determine whether BTZ@HMSNs can affect the migration and invasion of SNK-1 cells. As shown in Figure 4, compared with cell treated with PBS or HMSNs, BTZ treatment curbed cell migration and invasion of SNK-1 cells (Figure 4, *p* < 0.05). More importantly, compared with cells with BTZ, cell migration and invasion were decreased in SNK-1 cells with BTZ@HMSNs, which further confirmed the anticancer effectiveness of BTZ@HMSNs (Figure 4, *p* < 0.05).

### Dual role of BTZ@HMSNs in SHP-1/c-Kit/NF-κB/JAK1 pathways in SNK-1 cells

**Figure 5:**
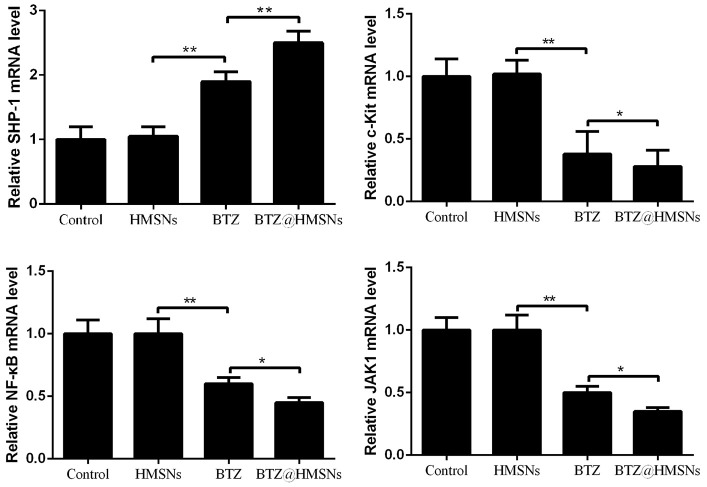
**The mRNA levels of Src homology 1 domain-containing protein tyrosine phosphatase (SHP-1), c-Kit, nuclear factor-κB (NF-κB), and JAK1 in SNK-1 cells treated with PBS (control), HMSNs, BTZ (75 nM), BTZ@HMSNs (containing 75 nM of BTZ) by RT-PCR.** BTZ, bortezomib; HMSNs, hollow mesoporous silica nanospheres. The differences between groups were analyzed by one-way ANOVA followed by multiple comparison with Tukey test. *P < 0.05 and **P < 0.01. Values are means ± SD.

To investigate the role of BTZ@HMSNs in suppression of lymphoma development, we assessed the transcriptome of cells treated with PBS, HMSNs, BTZ or BTZ@HMSNs. Through RT-PCR assay, we found that compared with cells treated with HMSNs, BTZ treatment increased the mRNA level of *SHP-1* (Figure 5). Reciprocally, the mRNA levels of *c-Kit*, *NF-κB*, and *JAK1* were suppressed by BTZ treatment (Figure 5, *p* < 0.01). Accordingly, the treatment of BTZ@HMSNs further increased the mRNA level of *SHP-1*, while reduced the mRNA levels of *c-Kit, NF-κB,* and *JAK1* (Figure 5, *p* < 0.05). Our results thus uncover BTZ@HMSNs elicit the opposite effects on SHP-1/c-Kit/NF-κB/JAK1 pathways in SNK-1 cells.

### BTZ@HMSNs restricts the development of lymphoma *in vivo*

**Figure 6:**
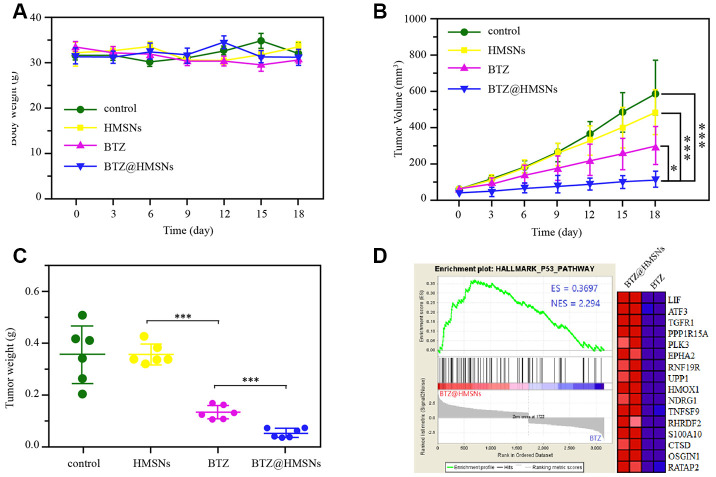
**BTZ@HMSNs improves anti-tumor effect of BTZ on lymphoma.** (**A**) The body weights of mice with different treatments, including PBS (control), BTZ (0.3 mg/kg), HMSNs, and BTZ@HMSNs (containing 0.3 mg/kg of BTZ), respectively, every three days for 18 days. (**B**) The tumor volumes of mice with different treatments every three days for 18 days. (**C**) The tumor weights of mice with different treatments on 18 days. (**D**) Gene set enrichment analysis (GSEA) of differentially expressed genes between the BTZ-treated tumors and BTZ@HMSNs-treated tumors. The differences between groups were analyzed by one-way ANOVA followed by multiple comparison with Tukey test. *P < 0.05 and ***P < 0.001. Values are means ± SD.

To further investigate the anticancer effects of BTZ@HMSNs *in vivo*, we first treated mice with PBS, BTZ, HMSNs or BTZ@HMSNs. As shown in Figure 6A, similar to the PBS and HMSNs, both BTZ and BTZ@HMSNs treatment hardly influenced the body weight of mice, suggesting that BTZ@HMSNs treatment is low toxic for normal tissues. We next subcutaneously injected Daudi cells into nude mice and ensued administration with PBS, BTZ, HMSNs or BTZ@HMSNs, respectively. Unlike the little anticancer effects of PBS or HMSNs treatment, the treatment of BTZ exhibited mild suppressive effects on the tumor growth and weight (Figure 6B, 6C, *p* < 0.05). In accordance to the data *in vitro*, compared with mild suppression by BTZ, BTZ@HMSNs significantly inhibited the development of lymphoma (Figure 6B, 6C, *p* < 0.05). Besides, we used RNAseq assay to analyze the transcriptome of tumors treated with BTZ or BTZ@HMSNs. Analogue to the activation of signaling related with apoptosis *in vitro*, the expression of genes related to p53 signaling pathway were selectively enhanced by the treatment of BTZ@HMSNs (Figure 6D). Our data thus demonstrate that BTZ@HMSNs can activate cell death pathway and suppress tumor growth *in vivo*.

## DISCUSSION

**Figure 7:**
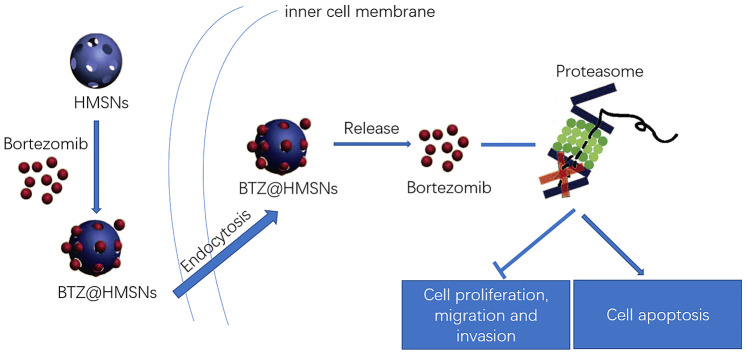
**Schematic illustration of the loading and delivery and release of BTZ to the cancer cells by HMSNs.**

In the present study, we successfully prepared BTZ@HMSNs with a persistent release of BTZ within 24 h. Compared with free BTZ, BTZ@HMSNs significantly diminished cell viability, migration and invasion as well as induced cell apoptosis in SNK-1 cells. Furthermore, BTZ@HMSNs obviously increased the mRNA level of SHP-1, as well as decreased the mRNA levels of c-Kit, NF-κB, and JAK1. These results indicated that BTZ@HMSNs might improve anti-cancer effects, and regulate SHP-1/c-Kit/NF-κB/JAK1 pathway in SNK-1 cells (Figure 7).

Due to poor water solubility, many hydrophobic anti-cancer drugs are clinically limited for cancer treatment [13]. The HMSNs have been reported to be able to overcome the application limitation of hydrophobic drugs because of its attractive properties, including large hollow interior, highly-hydrophilic nature and excellent biocompatibility [17]. Herein, BTZ as a hydrophobic anti-cancer drug was loaded into the HMSNs in order to improve drug deliver. This study revealed that almost 60 % of BTZ could be released from HMSNs within 10 h, and about 30% of BTZ was released within 24 h with a sustained and slow release, which was consistent with previous study [19]. These results suggested the sustained release property of BTZ@HMSNs, and increased drug accumulation might be able to decrease toxic adverse effect as well as improve the long-term chemotherapy efficacy for cancer cells [20].

BTZ is considered as an effective proteasome inhibitor, and BTZ-based therapy has been proved to be successfully applied to the clinical treatment of mantle cell lymphoma [21], diffuse large B-cell lymphoma [8], and plasmablastic lymphoma [9]. The occurrence and progression of cancers are closely related to several key cell biological processes, such as cell proliferation, apoptosis, migration and invasion. In this study, individual HMSNs treatment had no adverse effects on cell proliferation, apoptosis, migration and invasion, which suggested the excellent biocompatibility and nontoxicity of HMSNs in SNK-1 cells. Notably, free BTZ treatment inhibited cell viability and clone number in SNK-1 cells, while the BTZ@HMSNs showed enhanced inhibiting effect on cell proliferation. Consistent with the results of cell proliferation, BTZ@HMSNs exhibited increased the number of apoptotic cells than free BTZ. It is well known that cell apoptosis is mainly regulated by mitochondria-mediated apoptosis pathway in cancers [22, 23]. As a downstream molecule of apoptosis pathway, caspase-3 can decrease the ratio of Bcl-2/Bax and contribute to cell apoptosis [23, 24]_._ Thus, the expression of apoptosis-related proteins (Bcl-2, Bax, and cleaved-caspase-3) was analyzed in this study, and found that BTZ@HMSNs further inhibited Bcl-2 expression as well as promoted the expression of Bax and cleaved-caspase-3. Moreover, this study revealed decreased cell migration and invasion induced by BTZ@HMSNs in SNK-1 cells. All these results indicated that the BTZ@HMSNs possessed enhanced anti-tumor effects than free BTZ treatment.

Furthermore, the potential mechanism of the anti-tumor effects of the BTZ@HMSNs were explored in this study. SHP-1 as a non-receptor protein tyrosine phosphatase plays key roles in maintaining protein tyrosine phosphorylation *in vivo* [25]. c-Kit is a receptor tyrosine kinase frequently mutated in cancers, and is considered as an oncogene [26, 27]. SHP-1 can regulate cell proliferation, apoptosis, adhesion and metastasis by down-regulating several growth factor receptors, such as c-Kit [28, 29]. In addition, SHP-1 also can dephosphorylate NF-κB and JAK proteins [30]. Plenty of evidences have suggested that NF-κB activation and JAK signaling pathway are involved in the occurrence and progression of cancers through mediating inflammatory response and immune response as well as cell proliferation, apoptosis, and metastasis [31, 32]_._ This study showed that BTZ@HMSNs further increased the mRNA level of SHP-1 as well as reduced the mRNA levels of c-Kit, NF-κB, and JAK1, which indicated that the anti-cancer effect of BTZ@HMSNs might be related to SHP-1/c-Kit/NF-κB/JAK1 pathway.

In conclusion, this study reported that BTZ@HMSNs showed improved tumor-suppressing effect on SNK-1 cells compared to free BTZ, which might be attributable to the multiple attractive properties, including highly-hydrophilic nature, excellent biocompatibility and sustained drug releasing. Therefore, HMSNs are promising drug delivery systems for delivering therapeutic agents to treat various cancers.

## MATERIALS AND METHODS

### Preparation of BTZ@HMSNs

In brief, 82.1 mg of Zn(NO_3_)_2_.6H_2_O and 297.4 mg of 2-methylimidazole were separately dissolved in 40 mL of anhydrous methanol, and then the two mixtures were stirred at 400 rpm for 30 min. After centrifuged at 11,000 rpm for 10 min, a white solid was obtained, and dried using a vacuum freeze dryer. Subsequently, 100 mg of the above granules were dissolved in 40 mL of water, and then 1.6 g of cetyltrimethylchlorohydrin and 400 μL of 0.1 mol/L NaOH solution were added in turn. Next, 150 μL of a 20% by volume solution of tetraethyl orthosilicate in methanol was added dropwise every 30 min for 6 times. The crude product was collected by centrifugation and washed alternately with water and ethanol for three times, followed by addition of 30 mL of 0.1 mol/L hydrochloric acid solution to make the solution clear from milky white. The emulsion was washed twice with absolute ethanol and ultrapure water, and dried by a vacuum freeze dryer to obtain HMSNs.

Afterwards, 20.0 mg of the synthesized HMSNs were mixed with 200 μL of BTZ/DMSO solution (10 mM, purchased from LC Laboratories, Woburn, MA, USA) and stirred for 48 h at room temperature. BTZ@HMSNs were collected, followed by centrifugation at 14,000 rpm for 10 min to remove unbound BTZ. Meanwhile, supernatants were collected to determine the amount of BTZ by UV-Vis spectroscopy at a wavelength of 270 nm. Loading capacity (LC) was calculated as following: LC% = Wt (weight of BTZ in HMSNs)/Ws (weight of freeze-dried HMSNs) × 100%. The morphologies of BTZ@HMSNs were observed by scanning electron microscope (SEM; S-4800; Hitachi, Tokyo, Japan) and transmission electron microscope (TEM; Tecnai G2 20 S-TWIN, FEI, Eindhoven, Netherlands). Dynamic light scattering (DLS) was performed to measure the size distribution of BTZ@HMSNs by Zetasizer Nano Z (Worcestershire, UK). The nitrogen (N2) absorption experiment was carried out using a Micromeritics ASAP 2020 system at 77 K. The pore size distribution was calculated using the desorption isotherm branch by the BJH method.

### *In vitro* drug release analysis

The BTZ@HMSNs were dispersed in PBS solution, and shaken in a 37° C constant temperature shaker at 200 rpm in the dark. Followed by centrifugation at 14,000 rpm for 10 min, the supernatant was separately taken at 0.5 h, 1 h, 2 h, 4 h, 6 h, 9 h, 12 h, 24 h, and 72 h. Lastly, the amount of BTZ released in the supernatant was measured by a spectrophotometer (U-3010; Hitachi) at a wavelength of 270 nm, and an *in vitro* release profile was calculated.

### Cell culture

Human NK cell lymphoma cell line SNK-1 and B cell lymphoma cell lines Raji and Daudi were obtained from Shanghai Obio Technology Co., Ltd (China), and T cell lymphoma cell line Jurkat and NK cell lymphoma cell line NK-92 were purchased from the American Type Culture Collection (ATCC). Cells were maintained in RPMI-1640 medium (Gibco, Carlsbad, CA, USA) containing 10% fetal bovine serum (Gibco) in absence or presence of 200 units/mL recombinant human IL-2 (SNK-1 and NK-92) under standard incubation conditions (5% CO_2_ and 37° C).

### MTT assay

SNK-1 cells were grown in 96-well plates for 24 h, and then incubated with PBS (as control), BTZ (set from 1 to 150 nM), HMSNs, and BTZ@HMSNs (containing 1-150 nM of BTZ), respectively, for 24 h. Next, MTT (10 μL, Sigma, St Louis, MI, USA) was added to incubate with cells for 4 h, and dimethyl sulfoxide (150 μL, Sigma) was then used to dissolve formazan precipitates. The zero hole (medium, MTT, DMSO) and blank hole were set up. The absorbances at 450 nm were read by microplate reader (Molecular Devices, USA).

### Colony formation assay

SNK-1 cells were grown in 6-well plates for 24 h, and then cultured with RPMI-1640 medium containing 10% FBS and PBS, BTZ (75 nM), HMSNs, or BTZ@HMSNs (containing 75 nM of BTZ) for 10 days. Next, 0.5% (w/v) crystal violet in ethanol was added into cells for 5 min. The mean number of colonies was calculated under 10 different fields of vision.

### Observation of cell death fluorescence

SNK-1 cells were grown in 96-well plates for 24 h, and then incubated with PBS, BTZ (75 nM), HMSNs, and BTZ@HMSNs (containing 75 nM of BTZ), respectively, for 24 h. Then the cells were stained with 10 mg/mL of Hoechst 33258 for 1 h and 100 mg/mL of propidium iodide (PI) for 30 min. Lastly, Operetta high content analysis system (PerkinElmer) was used to observe the fluorescence.

### Cell apoptosis assay

Annexin V-FITC Apoptosis Detection kit was used to evaluate the cell apoptosis. SNK-1 cells were treated with PBS, BTZ (75 nM), HMSNs, and BTZ@HMSNs (containing 75 nM of BTZ), respectively, for 24 h. Next, cells were digested with Trypsin and washed with PBS, followed by resuspending in 1 × Binding Buffer, and stained with PI and FITC-Annexin V for 15 min at 25° C in the dark. Cells were finally detected using flow cytometer (Beckman Coulter, Fullerton, CA, USA).

### Western blotting assay

SNK-1 cells were treated with PBS, BTZ (75 nM), HMSNs, and BTZ@HMSNs (containing 75 nM of BTZ), respectively, for 24 h. Total proteins were obtained using lysis buffer, and then quantitated by bicinchoninic acid kit (Beyotime, Shanghai, China). Following sample separating and transferring into PVDF membranes, membranes were immerged in 5% nonfat milk for 1 h. Next, primary antibodies of Bcl-2, Bax, and cleaved-caspase-3 (1: 800, Sigma), as well as β-actin (1: 1000, Beyotime), respectively, were used for immunoblotting of the membranes overnight at 4° C. Then, membranes were reacted with secondary antibody (1: 1000, Beyotime) for 2 h keeping in dark place at room temperature. The signals were revealed using enhanced chemiluminescence Plus reagent (Beyotime) to image blots. The band quantification was carried out using Image J software.

### Transwell assay

Transwell inserts (Corning, New York, NY, USA) was used to detect cell migration and invasion abilities. Firstly, SNK-1 cells were plated onto the upper compartment coated with Matrigel containing medium free of serum, and then treated with PBS, BTZ (75 nM), HMSNs, and BTZ@HMSNs (containing 75 nM of BTZ), respectively, for 24 h. Meanwhile, complete 1440 medium was added into the bottom compartment for 24 h. Next, the cells in the bottom compartment insert was fixed with 95% ethanol, and then stained with crystal violet for 5 min. Five visual fields of × 200 magnification of each insert were randomly selected, and the number of migrated or invaded cells was calculated by an inverted microscope (Olympus, Japan).

### qRT-PCR

SNK-1 cells were treated with PBS, BTZ (75 nM), HMSNs, and BTZ@HMSNs (containing 75 nM of BTZ), respectively, for 24 h. Next, total RNA from cells was obtained by Trizol (Invitrogen), and then reverse transcription of RNA was performed using PrimeScript™ RT reagent Kit (Takara, Dalian, China). The qRT-PCR was carried out by the SYBR Premix Ex Taq TM II (Takara) on Rotor-Gene RG-3000A (Corbett Life Science, Sidney, Australia). The sequences of gene primers are listed in Table 1. GAPDH were served as the internal control, and data were analyzed with 2^-ΔΔCt^ method.

**Table 1:** Primers used for the qRT-PCR.

**Gene**	**Primer sequence**
SHP-1	F:5'-GGTCACCCACATCAAGGTCAT-3'
	R: 5'-TGTCGAAGGTCTCCAAACCAC-3'
c-Kit	F: 5'-CGAGATTAGGCTGTTATGC-3'
	R: 5'-ATCCATTCATTCTGCTTATTCT-3'
NF-κB	F: 5'-TGCAGAAAGAAGACATTGAGGTG-3'
	R: 5'-AGGCTAGGGTCAGCGTATGG-3'
JAK1	F: 5'-CGCTCTGGGAAATCTGCT-3'
	R: 5'-TGATGGCTCGGAAGAAAGG-3'
GAPDH	F: 5'-AGAAGGCTGGGGCTCATTTG-3'
	R: 5'-AGGGGCCATCCACAGTCTTC-3'
IL2RA	F: 5'-GCAATTTCGCCGTTGAAGAG-3'
	R: 5'-TAGGGTGGAGAGAGTTCCATAC-3'
CD3e	F: 5'-CAGAGGAAGCAAACCAGAAGA-3'
	R: 5'-GTGATGCAGATGTCCACTATGA-3'
CD19	F: 5'-AGCTGTGACTTTGGCTTATCT-3'
	R: 5'-GGGTCAGTCATTCGCTTTCT-3'
CD20	F: 5'-TGTGTTGTCACGCTTCTTCT-3'
	R: 5'-GCCTATCCAAGGAACAGGTTAG-3'
NCAM1	F: 5'-GCTTTGGAGGTGGAACTCTATT-3'
	R: 5'-ATGCTGGGTAGGGATGTTAATG-3'
EBV-LMP1	F: 5'-CTATTCCTTTGCTCTCATGC-3'
	R: 5'-TGAGCAGGAGGGTGATCATC-3'

### Animal model and treatments

Approval from the animal Ethics Committee of Centre Hospital of Cangzhou was obtained prior to experiments. All animal experiments were carried out in accordance with the National Institutes of Health guide for the care and use of Laboratory animals. Athymic (nude) mice (weighting 18-22 g, purchased from Charles River, Beijing, China) were used for the following experiments after one week of acclimation. To obtain the lymphoma mice model, 3 × 10^6^ of Daudi cells (a burkitt lymphoma cell line) per mouse were inoculated subcutaneously in mice. Then, lymphoma mice were treated with PBS, BTZ (0.3 mg/kg), HMSNs, and BTZ@HMSNs (containing 0.3 mg/kg of BTZ) via rapid tail vein injection, respectively. The body weight, and tumor volume of mice were monitored every three days for 18 days. On day 18, mice were euthanized, and the tumor was resected for the measurement of tumor weight.

### Statistical analysis

SPSS Statistics 20.0 software (IBM, Armonk, NY, USA) was used for data statistical analysis. Data were expressed as the mean ± SD. The differences between groups were analyzed by one-way ANOVA followed by multiple comparison with Tukey test. *P* value < 0.05 indicated statistically significant.
